# Gigantic negative magnetoresistance in the bulk of a disordered topological insulator

**DOI:** 10.1038/ncomms15545

**Published:** 2017-05-25

**Authors:** Oliver Breunig, Zhiwei Wang, A A Taskin, Jonathan Lux, Achim Rosch, Yoichi Ando

**Affiliations:** 1 Physics Institute II, University of Cologne, Zülpicher Strasse 77, 50937 Köln, Germany; 2 Institute for Theoretical Physics, University of Cologne, Zülpicher Strasse 77, 50937 Köln, Germany

## Abstract

With the recent discovery of Weyl semimetals, the phenomenon of negative magnetoresistance (MR) is attracting renewed interest. Large negative MR is usually related to magnetism, but the chiral anomaly in Weyl semimetals is a rare exception. Here we report a mechanism for large negative MR which is also unrelated to magnetism but is related to disorder. In the nearly bulk-insulating topological insulator TlBi_0.15_Sb_0.85_Te_2_, we observed gigantic negative MR reaching 98% in 14 T at 10 K, which is unprecedented in a nonmagnetic system. Supported by numerical simulations, we argue that this phenomenon is likely due to the Zeeman effect on a barely percolating current path formed in the disordered bulk. Since disorder can also lead to non-saturating linear MR in Ag_2+*δ*_Se, the present finding suggests that disorder engineering in narrow-gap systems is useful for realizing gigantic MR in both positive and negative directions.

## Abstract (web summary)

Large negative magnetoresistance is usually related to magnetism and the exceptions are rare. Here, Breunig *et al*. report a large negative magnetoresistance in a topological insulator, TlBi_0.15_Sb_0.85_Te_2_, which is likely due to the Zeeman effect on a barely percolating current path formed in the disordered bulk.

Magnetic fields tend to localize electrons by trapping them in cyclotron orbits, leading to positive magnetoresistance (MR) in metals and semiconductors. Large negative MR is therefore unusual and is always a signature of some peculiar physics. So far, three mechanisms are widely known to cause large negative MR: quenching of the Kondo effect^1^,^2^, magnetic-field-induced phase transition from a paramagnetic insulator to a ferromagnetic metal (so-called colossal MR)^3^, and the chiral anomaly in Weyl semimetals^4^,^5^,^6^,^7^. The last one is expected to be observed only in the longitudinal configuration (that is, when the current is parallel to the magnetic field) and has been of significant interest ever since three-dimensional (3D) Dirac semimetals and Weyl semimetals started to attract attentions^8^,^9^. Intriguingly, large negative, longitudinal MR of similar character has been observed in materials having no Weyl nodes^10^, and the meaning of negative MR in nonmagnetic systems is currently being scrutinized^11^,^12^. In this context, the finding of a new mechanism for large negative MR in zero-gap or narrow-gap systems without magnetism would be important for establishing a general understanding of the negative MR. This paper reports an unexpected discovery of a gigantic negative MR in a nearly bulk-insulating topological insulator (TI), which turned out to be a platform to realize large negative MR in the bulk through a new mechanism related to disorder.

The key feature of 3D TIs is the topologically protected surface states^13^,^14^, and significant efforts have been devoted to find ways to achieve sufficiently bulk-insulating samples to address the transport properties through the surface states^14^,^15^. The most successful strategy to achieve this goal has been to adopt the concept of compensation, namely, balancing the numbers of donors and acceptors^16^. This has led to the successful achievements of surface-dominated transport in bulk single crystals of Bi_2−*x*_Sb_*x*_Te_3−*y*_Se_*y*_ (BSTS)^17^. We have recently synthesized another solid-solution system of 3D TIs, TlBi_*x*_Sb_1−*x*_Te_2_ (TBST), which belongs to the family of thallium-based ternary III–V–VI_2_ chalcogenides^18^,^19^,^20^,^21^,^22^ and the chemical potential can be tuned through the bulk band gap between *n*- and *p*-type states^23^. We found that the maximal compensation in TBST is achieved near *x*≃0.2 (see Supplementary Note 1 for details), where one can obtain nearly bulk-insulating samples. The gigantic MR is discovered in such a compensated sample of TBST.

As we show with theoretical simulations, in compensated TI systems the spatially fluctuating Coulomb potential caused by charged donors and acceptors leads to the appearance of charge puddles, which can provide percolating current paths if the compensation is not perfect; furthermore, such current paths are greatly affected by applied magnetic fields due to the Zeeman effect, resulting in an increased conductivity in high magnetic fields. This leads to an essentially isotropic, gigantic negative MR, and both the magnitude and the magnetic-field dependence observed in experiments are well reproduced by theoretical simulations involving a random resistor network. The agreement between theory and experiment strongly suggests that disorder can be a key player in the phenomenon of large negative MR.

## Results

### Sample characterizations

**Figure 1:**
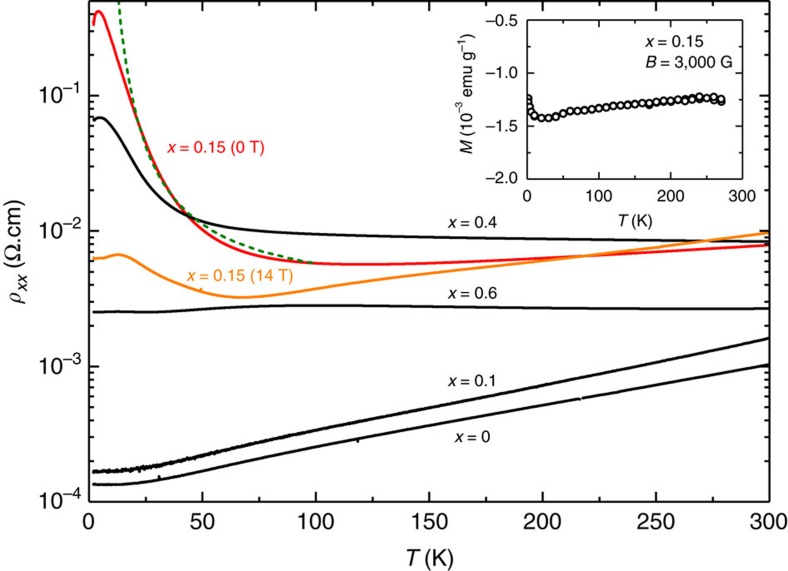
Resistivity behaviour of TBST. The temperature dependences of the resistivity, *ρ*_*xx*_(*T*), in the *ab* plane are plotted for *x*=0, 0.1, 0.15, 0.4 and 0.6 in 0 T; the green dashed line depicts a simple activated behaviour with an activation energy of 6 meV. The data in 14 T are additionally shown for *x*=0.15 (orange line). Inset shows the magnetization data for *x*=0.15.

**Figure 2:**
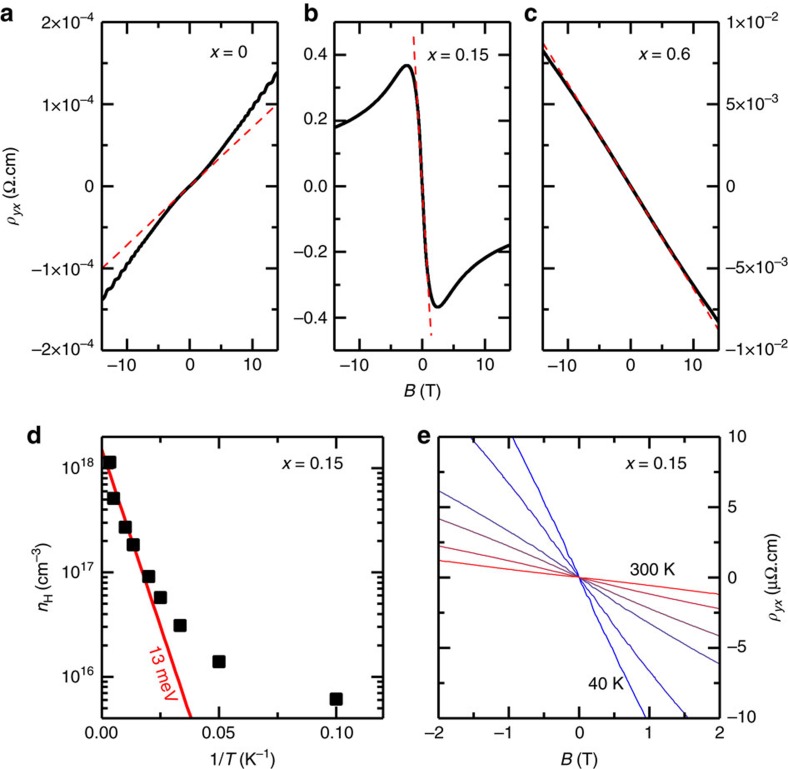
Hall effect in TBST. (**a**–**c**) The magnetic-field dependences of the Hall resistivity, *ρ*_*yx*_(*B*), for *x*=0, 0.15, and 0.6 measured at 2 K; the red dashed lines represent the low-field slope to yield an estimate of the densities of holes (*p*) and electrons (*n*), which are (**a**) *p*=8 × 10^19^ cm^−3^, (**b**) *n*=1.9 × 10^15^ cm^−3^ and (**c**) *n*=1 × 10^18^ cm^−3^. The strongly changing slope in **b** might prompt a two-band analysis, but the gigantic negative MR clearly negates its applicability; according to our puddle scenario, the non-linear *ρ*_*yx*_(*B*) comes from a change in the number of mobile bulk carriers with *B*. (**d**) Arrhenius plot of the Hall carrier density *n*_H_ for *x*=0.15, giving an approximate activation energy of 13 meV (red line). (**e**) *ρ*_*yx*_(*B*) data for *x*=0.15 measured at *T*=300, 200, 100, 75, 50 and 40 K.

Figure 1 shows the temperature dependences of the resistivity *ρ*_*xx*_ in TBST for various *x* values. Among the samples showing metallic behaviour, *x*=0 and 0.1 are *p*-type, while *x*=0.6 is *n*-type (see Fig. 2a,c for the Hall data). A slight increase in *x* from 0.1 to 0.15 causes *ρ*_*xx*_ at ∼10 K to increase by three orders of magnitude to 0.4 Ωcm, and an insulating behaviour (*∂ρ*_*xx*_/*∂T*<0) arises between 10 to 100 K. We focus on this *x*=0.15 sample, in which the Hall carrier density *n*_H_ extracted from the zero-field slope of the Hall resistivity *ρ*_*yx*_(*B*) (Fig. 2b) is only 1.9 × 10^15^ cm^−3^ at 2 K and approximately follows the Arrhenius law with an activation energy Δ_*μ*_ of 13 meV (Fig. 2d). The compensation in TBST is not as effective as that in BSTS^17^ and the maximum attainable resistivity is lower; consequently, we found no evidence for a surface contribution to the transport properties.

### Magnetoresistance

**Figure 3:**
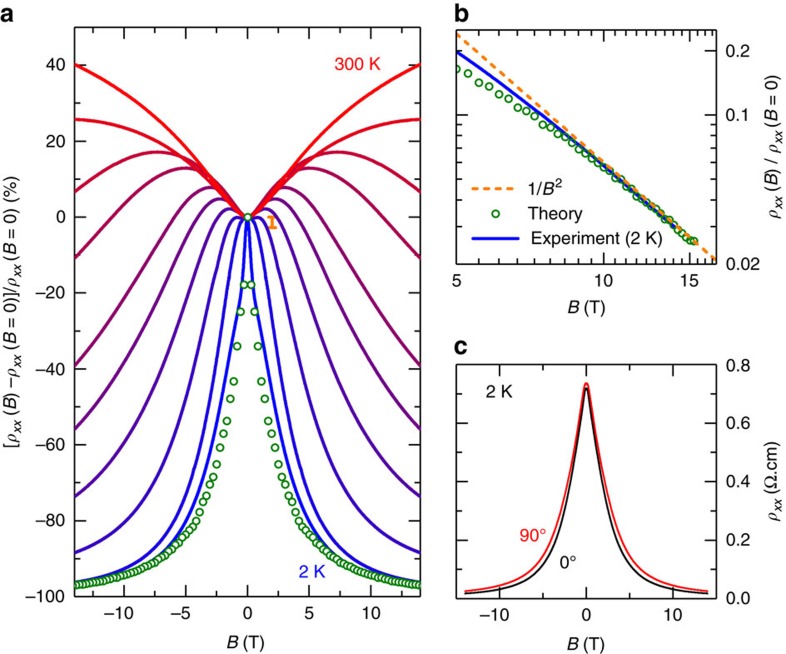
Magnetoresistance in TlBi_0.15_Sb_0.85_Te_2_ for magnetic fields along the *c* axis. (**a**) The change in the resistivity *ρ*_*xx*_ with magnetic field *B*, normalized by *ρ*_*xx*_(*B*=0), at different constant temperatures *T*=300, 200, 100, 75, 50, 40, 30, 20, 10 and 2 K from top (red) to bottom (blue); open green circles are obtained by numerically solving a resistor network for the disordered Coulomb system at *T*=0 K as explained in the main text. The vertical bar at 2 T shows an estimate of the possible 3D weak localization contribution^24^. (**b**) Double-logarithmic plot of *ρ*_*xx*_(*B*)/*ρ*_*xx*_(*B*=0) showing that the low-*T*/ high-*B* data approach a 1/*B*^2^ dependence indicated by a dashed orange line; the numerical results (open green circles) also approach the 1/*B*^2^ behaviour at high *B*. (**c**) *ρ*_*xx*_(*B*) data for two different magnetic field orientations, *B*||*c* and 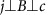; here, the *c* axis is along the [111] direction of the rhombohedral unit cell and is normal to the crystallographic layers.

The surprising finding is that the magnetic field of 14 T leads to a reduction of *ρ*_*xx*_ at 10 K by nearly two decades from 400 to 6.5 mΩcm (orange line in Fig. 1). How this gigantic negative MR evolves with temperature is shown in Fig. 3a; with decreasing temperature, the MR changes from entirely positive at ≳200 K to entirely negative at ≲20 K. The additional cusp-like feature seen at 2 K for *B*≲1 T may be attributed to a weak localization effect, but it cannot explain the large negative MR; this can be seen by the estimate of the possible 3D weak localization contribution^24^ for 2 T shown in Fig. 3a (see Supplementary Note 2 for details). The logarithmic plot of *ρ*_*xx*_(*B*) shown in Fig. 3b suggests that the MR behaviour approaches a 1/*B*^2^ dependence in high fields at low temperature.

It is important to note that the compositional analysis using inductively coupled plasma atomic-emission spectroscopy found no magnetic impurities in TBST, which is corroborated by the magnetic susceptibility data (inset of Fig. 1). Hence, magnetism is not involved in this gigantic negative MR. Also, the chiral anomaly is irrelevant, because the negative MR shows up in the transverse configuration. Its origin is probably not related to the anisotropy in the band structure, because the difference between *B*||*c* and 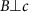 is negligible (Fig. 3c).

Phenomenologically, the appearance of the negative MR in TBST is tied to the emergence of an insulating behaviour. In fact, among the investigated samples, all those that show an insulating behaviour (0.13≤*x*≤0.4) presented a negative MR which was most pronounced in the *x*=0.15 sample shown here (additional data for other *x* values are shown in the Supplementary Fig. 1). The onset temperature of the negative MR on cooling compares well with the onset of an activated behaviour in *ρ*_*xx*_(*T*). A fit of the data below 100 K to 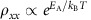, shown as a dashed green line in Fig. 1, yields an activation energy *E*_A_≈6 meV. This value is substantially smaller than the bulk band gap of ∼0.1 eV (refs 22, 23). Such a small effective activation energy in compensated TIs (refs 15, 16) has been discussed to be a signature of the formation of charge puddles^25^,^26^,^27^, which we discuss in the following to be responsible for the gigantic negative MR.

### Electron puddles

The concept of electron and hole puddle formation has been adopted in the framework of compensated TIs to explain their small effective activation energy^25^,^26^,^27^ as well as the unusual temperature dependence of the bulk optical conductivity^28^. Basically, the strong fluctuations of the Coulomb potential introduced by positively charged empty donors and negatively charged occupied acceptors, which coexist in compensated systems, inevitably create regions where the conduction-band bottom or the valence-band top crosses the chemical potential, leading to the appearance of locally conducting puddles containing either electrons or holes^29^.

Similar to other compensated TIs, the impurity states in TBST are shallow due to a rather large dielectric constant 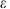
≈200, approximated from that of TlSbTe_2_ (ref. 30). Thus, their energies are close to the edge of the conduction or valence band. The *x*=0.15 sample is *n*-type and is slightly away from complete compensation, leading to the pinning of the chemical potential at low to moderate temperature to the donor impurity levels, which lie below but close to the conduction band bottom. The obtained activation energy Δ_*μ*_=13 meV corresponds directly to *E*_0_−*μ*, which is the energy difference between the conduction band bottom in 0 T, *E*_0_, and the chemical potential *μ*. The dominance of a single *n*-type channel at moderate temperature is also indicated by the linear *ρ*_*yx*_(*B*) behaviour at *T*≥40 K (Fig. 2e).

At low temperature where the screening due to thermally activated carriers is negligible, the fluctuations of the Coulomb potential arising from charged impurities pile up. In this situation, the same mechanism as in the case of electron-hole puddles^27^ enforces the formation of spatially separated electron puddles. Skinner *et al*.^27^ pointed out that one obtains effectively a reduced activation energy in the presence of strong fluctuations of the Coulomb potentials. Hence, the activation energy *E*_A_ (≈6 meV) observed in the low-temperature *ρ*_*xx*_(*T*) data is actually expected to differ from Δ_*μ*_ extracted from the *n*_H_(*T*) behaviour at higher temperature.

Below 4 K, the *ρ*_*xx*_(*T*) behaviour for *x*=0.15 is metallic; this is different from the prediction for a compensated TI in ref. 27, where a variable range hopping transport between puddles was postulated. Furthermore, in the case of hopping conduction, a positive MR would be expected due to the decreasing size of the atomic orbitals in strong magnetic fields^31^. The metallic behaviour indicates that either the electron puddles in the slightly doped samples weakly percolate or another conduction mechanism (for example, via impurity bands) connects them.

### Numerical simulations of puddles

Based on the above picture of the spatially disordered electronic states caused by electron puddles, we propose that the increasing conductivity in applied magnetic fields is a result of an increase of percolating puddles due to the Zeeman energy. To understand this effect, we performed numerical simulations of an imperfectly compensated Coulomb system. The characteristic energy scale of such a system is given by the Coulomb energy between neighbouring dopants 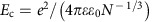 (ref. 28), where *N* denotes the dopant density. Since the dielectric constant in TBST is large (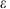
≈200 (ref. 30)) as discussed above, the temperature scale *E*_c_/*k*_B_ becomes small and we use this parameter to adjust the numerical results to the experimental data. Also, as the negative MR is most pronounced at low temperature, we restrict the analysis to the case 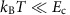. For the simulation, the degree of compensation was set at *K*=*N*_A_/*N*_D_=0.9–0.95, where *N*_A_ and *N*_D_ denote the densities of acceptors and donors, respectively. This implies that the chemical potential is close to the conduction band edge and that puddles are almost exclusively formed by electrons. The electron puddles are spatially confined to regions where the position-dependent electrostatic potential *φ*(**r**) (which is responsible for the local band bending) exceeds *E*_0_−*μ*; in other words, electron puddles appear when the local band bending *φ*(**r**) brings down the conduction band edge below the chemical potential.

Assuming a single spherical band with effective mass m*, the typical density of charges delocalized within the puddles at low temperature is given by


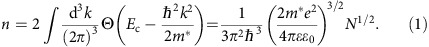


For 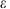
=200, m*=0.2*m*_e_ and *N*=3 × 10^20^ cm^−3^, we find a density *n*≈10^17^ cm^−3^, which is significantly smaller than that of the dopants, that is, 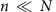. Thus, the impurity states serve as a reservoir for the delocalized states and approximately fix the chemical potential.

**Figure 4:**
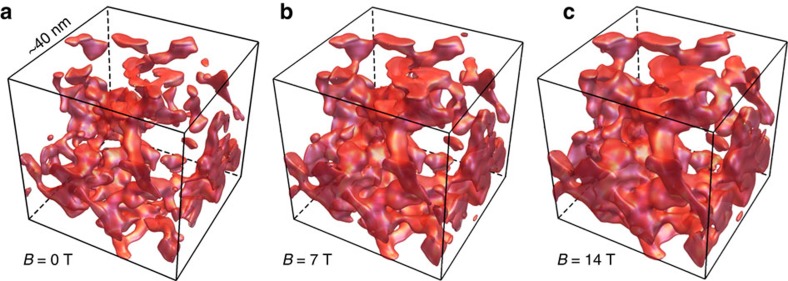
Simulations of electron puddles in magnetic fields. Spatial distribution of electron puddles in an imperfectly compensated Coulomb system at temperature *T*=0 K, simulated for the *g*-factor 6, characteristic Coulomb energy *E*_c_=4.9 meV, and the degree of compensation *K*=0.95. The coloured surfaces represent where the spatially fluctuating Coulomb potential *φ*(**r**) becomes equal to (*E*_0_−*μ*)−*E*_Z_(*B*), which is the criterion for the puddle formation (*E*_0_ is the energy of the conduction-band bottom in 0 T, *μ* is the chemical potential, and *E*_z_ is the Zeeman energy). As shown for the magnetic field *B* of (**a**) 0 T, (**b**) 7 T, and (**c**) 14 T, the volume of the enclosed regions increases with *B* due to the effect of the Zeeman energy. The chemical potential is assumed to be constant due to the pinning by the impurity levels, which work as a reservoir of delocalized carriers. Simulations are shown for a cube with a width of 40 nm corresponding to ∼3 × 10^4^ dopants. We have checked that the qualitative behaviour does not change in larger systems.

In such a situation, the volume accessible by the delocalized states (that is, the volume occupied by the puddles) increases with increasing magnetic field *B* due to the Zeeman band shift. Adopting *g*≈6 from a similar system TlBiSSe (ref. 32), the down shift of the band bottom, *gμ*_B_*B*, is estimated to be 0.35 meV T^−1^. This effect is visualized in Fig. 4, which shows a typical simulation result for *K*=0.95 (details are explained in the Methods section). Here we plot the surface contours where the local electrostatic potential supports puddle formation, that is, where *φ*(**r**)=(*E*_0_−*μ*)−*E*_Z_(*B*) is satisfied [*E*_Z_(*B*)=*gμ*_B_*B* is the Zeeman energy], for *E*_Z_/*E*_c_=0, 0.5, and 1, corresponding to *B*≃0, 7, and 14 T, respectively. Note that, due to the pinning of the chemical potential to the impurity levels, *E*_0_−*μ* is expected to be unchanged with *B*. Within the enclosed regions (that is, electron puddles), delocalized states exist. One can clearly see that with increasing magnetic field, previously disconnected puddles merge. Note that the occupation of most impurity states, and therefore the profile of the potential fluctuations, is assumed not to change with magnetic field. The increase in the percolating paths in *B*>0 demonstrated in Fig. 4 naturally leads to an increase in the electrical conductivity.

### Numerical simulations of magnetotransport properties

To quantify this effect we have calculated the conductivity at different magnetic fields using a random resistor network^33^ (details are explained in the ‘Methods' section). The local conductivity of each resistor in this network was calculated according to *σ*(**r**)=*μ*_e_*n*(**r**), where we have assumed a constant electron mobility *μ*_e_. For the calculation of the density *n*(**r**) we have assumed a locally free electron gas which gives 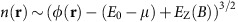 and *σ*(**r**)=0 if *φ*(**r**)<(*E*_0_−*μ*)−*E*_Z_(*B*). Note that this approach is tailored to describe the regime where puddles overlap and start to percolate. In this regime, variable range hopping effects^27^ (not included in our resistor network) can be neglected. Furthermore, weak localization effects, which we have already shown to be small, are not included. By fitting the numerical results at *B*=0 to the experimental data, we find a mobility of *μ*_e_≈11,500 cm^2^ V^−1^ s^−1^ corresponding to a scattering rate of *τ*^−1^≈10^12^ s^−1^, which is in reasonable agreement with the scattering rates measured in similar systems, see ref. 28 for an overview. These numbers depend, however, sensitively on *K* and therefore are only an order of magnitude estimate. Furthermore, one should note that this number only characterizes the typical mobility within a puddle, not the effective mobility obtained by averaging over the bulk. The shape and width of *ρ*_*xx*_(*B*)/*ρ*_*xx*_(*B*=0) depend only weakly on *K*, while *E*_c_ determines the width of the curve and is used as an adjusting parameter; we find *E*_c_=60±10 K reproduces the data well for *g*=6. We show in Fig. 3a,b our numerical results for *ρ*_*xx*_(*B*)/*ρ*_*xx*_(*B*=0) with *K*=0.9 and *E*_c_=56 K, which correspond to a dopant density *N*=3 × 10^20^ cm^−3^ and is in reasonable agreement with studies of similar systems^28^. Thus, a set of reasonable parameters reproduces the observed negative MR in our random resistor network simulation. Remarkably, our numerics even reproduces the approximate 1/*B*^2^ asymptotics of *ρ*_*xx*_ for high fields, see Fig. 3b. In even higher fields, when either a large volume fraction of the sample becomes a good metal or when our approximation of a fixed chemical potential breaks down, we expect a modified high-field asymptotics.

## Discussions

The above mechanism for the MR is expected to be independent of whether the configuration is transverse or longitudinal. Indeed, as is shown in the Supplementary Fig. 2, essentially the same negative MR is observed in the longitudinal configuration (see Supplementary Note 3 for details). As for the temperature dependence, although our numerical calculations were performed only for 0 K, it is naturally expected that the random Coulomb potential is gradually screened with increasing temperature by thermally activated carriers, which results in smearing of the puddles; consequently, the negative MR is gradually diminished. The positive MR observed at high temperature is relatively large and would be an interesting topic for future studies. It is prudent to mention that large negative MR has not been observed in other compensated TI materials such as BSTS. Our numerical simulations show that for the occurrence of the large negative MR, it is important that the parameter *K* is at a right value away from 1.0, so that the compensation is not perfect and the puddles of only one carrier type are weakly percolating in 0 T. Obviously, such a situation is not realized in BSTS, in which the degree of compensation is better than that in the present TBST series. We also note that the density of naturally created vacancies in the crystals may widely vary among different compounds, resulting in significantly different landscape/density of puddles and hence a different percolation behaviour.

As our simulations suggest, the gigantic negative MR in TlBi_0.15_Sb_0.85_Te_2_ is likely due to a new mechanism related to the spatial disorder of the electronic system in the form of puddles, which results in magnetic-field-sensitive percolation of the current paths. In this regard, it is interesting that large positive, non-saturating linear MR has been shown to originate from spatially distorted current paths in Ag_2+*δ*_Te and Ag_2+*δ*_Se (ref. 34), which are narrow-gap semiconductors and are potentially TIs (ref. 35); such distorted current paths could lead to negative MR, but only for the longitudinal configuration^36^. Obviously, disorder in narrow-gap systems plays a key role in both positive and negative gigantic MR, and its understanding may help to engineer practical devices for magnetic data storage and sensors.

## Methods

### Sample preparations

Single crystals of TBST were grown from a melt of high-purity (99.9999%) starting materials of elementary Tl, Bi, Sb and Te shots that were cleaned to remove the oxide layers formed in air^37^. They were mixed in the target composition and sealed in evacuated quartz tubes. After heating to 600 °C for 48 h, at which the tubes were intermittently shaken to ensure homogeneity of the melt, they were slowly cooled down to 400 °C in 100 h. Typically the growth resulted in multiple intertwined crystallites whose size increases from bottom of the grown boule to the top, and single crystals suitable for further analysis were prepared by cleavage at the top part. The actual chemical composition of the samples was confirmed to be consistent with the nominal one by inductively coupled plasma atomic-emission spectroscopy as well as by energy-dispersive X-ray spectroscopy. The crystal structure was confirmed by powder X-ray diffraction to be unchanged from both endmembers^38^ over the whole range of *x*.

### Measurements

Transport measurements were performed on thin needle-like samples with a typical dimension of 4 × 1 mm^2^ in the *ab* plane and 0.4 mm in the *c* axis. Using a bath cryostat equipped with a variable-temperature insert the resistivity *ρ*_*xx*_ and the Hall resistivity *ρ*_*yx*_ were measured simultaneously in sweeping magnetic fields (±14 T) with a low-frequency lock-in technique. Electrical contacts were prepared in a standard six-probe configuration using 25 μm platinum wires attached with dissolved silver paint that was cured at room temperature.

### Numerical simulations

To simulate the behaviour of the puddles in a magnetic field we have used the common static model of shallow donors and acceptors in compensated semiconductors^26^,^27^,^28^. The donors with density *N*_D_ and the acceptors with density *N*_A_=*KN*_D_ are placed at random, uncorrelated positions. Their bare energies are separated by a band gap Δ which was chosen as 15 × *E*_c_ in the simulations.

For a specific realization of the dopant positions (say **r**_1_, **r**_2_, …), the Hamiltonian reads


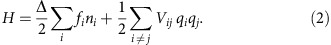


*q*_*i*_ denotes the charge in units of the elementary charge of the dopant at position **r**_*i*_. It can be either 0 or −1 for acceptors (*f*_*i*_=−1) and either 0 or +1 for donors (*f*_*i*_=+1). The occupation *n*_*i*_ of the *i*-th dopant is related to its charge *q*_*i*_ by *q*_*i*_=(*f*_*i*_+1)/2−*n*_*i*_.

The Coulomb interaction *V*_*ij*_ between the dopants is equipped with a short distance cutoff of the order of the effective Bohr radius 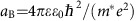, where m* denotes the effective mass. This accounts for the finite extent of the dopant wavefunctions^26^,^27^. With this approximation the effective interaction reads 
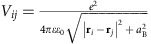
. The model is valid for 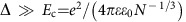 (otherwise one has to include the band states) and 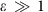 to ensure that the dopants are shallow.

We have performed simulations for zero temperature. Only small temperature effects are expected as long as 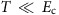 (ref. 28). To find the true groundstate is an exponentially hard problem, but there is an algorithm to find an approximate groundstate, called a pseudo-groundstate, in polynomial time^27^,^31^. The physical properties of a pseudo-groundstate are expected to be indistinguishable from that of the true groundstate.

We introduce the single electron energies as 
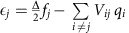
. A pseudo-groundstate is found when 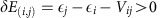 is satisfied for all proper pairs with *n*_*j*_=0 and *n*_*i*_=1. Simulations are performed in a cubic volume *V*=*L*^3^ with periodic boundary conditions. Once we have found a pseudo-groundstate, we plot the surface contour of constant potential as explained in the main text, where a typical result is shown.

For the calculation of the conductivity we proceed as follows once a pseudo-groundstate is found: The simulation volume is discretized and the grid points serve as nodes for the resistor network. We calculate the local conductivity between two neighbouring grid points from the Coulomb potential and the Zeeman energy as explained in the main text. The emerging resistor network is solved exactly, see ref. 33 for details on the algorithm (however, in contrast to ref. 33, we use physical boundary conditions where the currents, rather than the potentials, are set to zero on the growing face of the network). In this way, the conductivity of the full system is found. The grid size was chosen as *N*^−1/3^, which is the typical length scale (∼1.5 nm for *N*=3 × 10^20^ cm^−3^) on which the potential changes. By using finer grids, we have checked that discretization effects are small (≲5% and well within the numerical error bars). The simulations have been performed for ≈4.1 × 10^5^ dopants with periodic boundary conditions. The orbital MR inside the puddles is neglected in the present calculations.

### Data availability

The data that support the findings of this study are available from the corresponding author upon request.

## Additional information

**How to cite this article:** Breunig, O. *et al*. Gigantic negative magnetoresistance in the bulk of a disordered topological insulator. *Nat. Commun.* **8,** 15545 doi: 10.1038/ncomms15545 (2017).

**Publisher's note**: Springer Nature remains neutral with regard to jurisdictional claims in published maps and institutional affiliations.

## Supplementary Material

Supplementary Information Supplementary Figures, Supplementary Notes and Supplementary References
