# Supplementary material for: Gigantic negative magnetoresistance in the bulk of a disordered topological insulator
Source: Nat Commun. 2017 May 25;8:15545. doi: 10.1038/ncomms15545 (PMC5458500; doi:10.1038/ncomms15545)
Supplement: Supplementary Information — Supplementary Figures, Supplementary Notes and Supplementary References [file ncomms15545-s1.pdf]

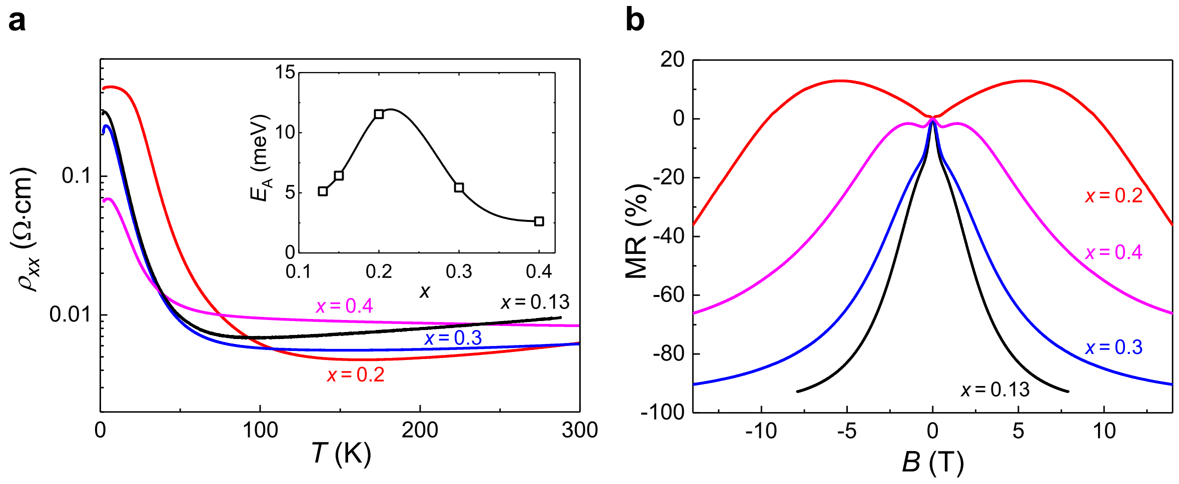

Supplementary Figure 1: **Transport data of $TlBi_xSb_{1-x}Te_2$ single crystals with various x values.** (**a**) Temperature dependencies of the resistivity ρ_{xx} in the ab plane for x = 0.13, 0.2, 0.3, and 0.4 in 0 T; inset shows the x dependence of the effective activation energy E_A extracted from the $\rho_{xx}(T)$ data (solid line is just a guide to the eye). (**b**) Magnetoresistance of the same set of samples in the transverse configuration (current $j \perp B \parallel c$) at 2 K.

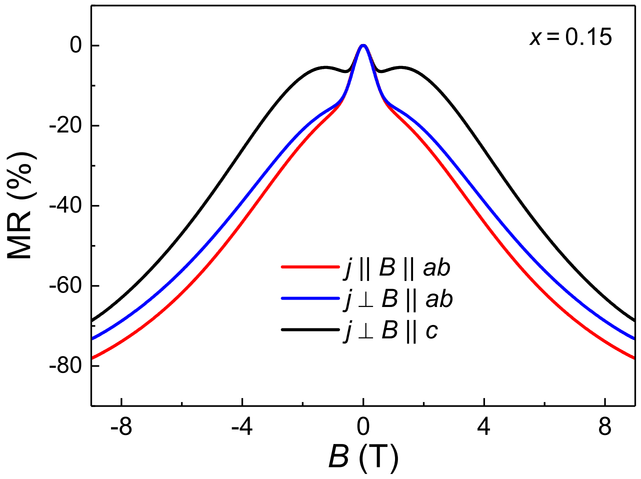

Supplementary Figure 2: **Magnetoresistance in various magnetic-field orientations.** Magnetoresistance (MR) of another $TlBi_{0.15}Sb_{0.85}Te_2$ sample at 2 K measured in three different configurations, longitudinal (current $j \parallel B \parallel ab$), transverse in-plane ($j \perp B \parallel ab$), and transverse out-of-plane ($j \perp B \parallel c$).

Supplementary Note 1: Resistivity and MR behavior at various x values.

As is mentioned in the main text, all the samples of $TlBi_xSb_{1-x}Te_2$ (TBST) that showed an insulating behavior ($0.13 \leq x \leq 0.4$) presented a negative magnetoresistance (MR), although the magnitude of the negative MR was smaller at x values other than 0.15. Supplementary Figure 1 shows the $\rho_{xx}(T)$ data and the MR data for $x = 0.13$, 0.2, 0.3, and 0.4. The inset of Supplementary Figure 1a shows the effective activation energy extracted from the upturn in $\rho_{xx}(T)$ at low temperature; this effective activation energy is an indicator of the degree of compensation and presents a maximum at $x = 0.2$. Interestingly, the negative MR at $x = 0.2$ is smaller than that at $x = 0.15$, which supports our interpretation that the gigantic negative MR requires the degree of compensation (quantified by the parameter K in the main text) to be at a right value away from 1.0.

Supplementary Note 2: Negative MR expected from 3D weak localization.

The TBST system studied here has a three-dimensional (3D) Fermi surface [1], and hence its transport should be treated as 3D. The magnetoconductance due to the weak localization effect in 3D systems has been calculated by Al'tshuler *et al.* [2] as

$$\Delta\sigma = \frac{e^2}{2\pi^2\hbar} f_3\left(\frac{B}{B_{\rm in}}\right)\left(\frac{eB}{\hbar}\right)^{1/2}, \tag{1}$$

where $B_{\rm in}$ is the characteristic magnetic field scale defined by the inelastic diffusion length L_ϕ via $B_{\rm in} = \hbar/(4\pi e L_\phi^2)$, and $f_3(x) = 0.605$ for $x \gg 1$. For a typical L_ϕ of 1 μm, $B_{\rm in}$ = 0.329 mT and one can safely replace $f_3(B/B_{\rm in})$ with 0.605 in the relevant magnetic-field range. We have calculated the negative magnetoresistance (MR) expected from the 3D weak localization by using the above formula, and the result for 2 T is shown as an example in the main text.

Supplementary Note 3: Comparison of transverse and longitudinal MR.

When the MR is caused by the Zeeman effect as is proposed in the main text, it should be essentially isotropic as long as the g factor is isotropic. We have performed experiments to make a direct comparison between transverse and longitudinal configurations using a $x = 0.15$ sample which is different from the one shown in the main text. As one can see in Supplementary Figure 2, the anisotropy in the MR behavior measured at 2 K in three different orientations of the magnetic field are all similar, supporting our interpretation that the MR behavior stems from the Zeeman effect.

Supplementary References

[1] Trang, C. X. *et al.* Metal-insulator transition and tunable Dirac-cone surface state in the topological insulator $TlBi_{1-x}Sb_xTe_2$ studied by angle-resolved photoemission. *Phys. Rev. B* **93**, 165123 (2016).

[2] Al'tshuler, B., Aronov, A. G., Larkin, A. I. & Khmel'nitskil, D. E. Anomalous magnetoresistance in semiconductors. *Sov. Phys. JETP* **54**, 411-419 (1981).
